# Autophagy and SARS-CoV-2-Old Players in New Games

**DOI:** 10.3390/ijms24097734

**Published:** 2023-04-23

**Authors:** Tsvetomira Ivanova, Yuliia Mariienko, Nikolay Mehterov, Maria Kazakova, Yordan Sbirkov, Krassimira Todorova, Soren Hayrabedyan, Victoria Sarafian

**Affiliations:** 1 Department of Medical Biology, Medical University-Plovdiv, 4000 Plovdiv, Bulgaria; 2 Research Institute, Medical University-Plovdiv, 4000 Plovdiv, Bulgaria; 3 Institute of Biology and Immunology of Reproduction, Bulgarian Academy of Sciences, 1113 Sofia, Bulgaria

**Keywords:** autophagy, SARS-CoV-2, treatment strategies

## Abstract

At present it is well-defined that autophagy is a fundamental process essential for cell life but its pro-viral and anti-viral role has been stated out with the COVID pandemic. However, viruses in turn have evolved diverse adaptive strategies to cope with autophagy driven host defense, either by blocking or hijacking the autophagy machinery for their own benefit. The mechanisms underlying autophagy modulation are presented in the current review which summarizes the accumulated knowledge on the crosstalk between autophagy and viral infections, with a particular emphasizes on SARS-CoV-2. The different types of autophagy related to infections and their molecular mechanisms are focused in the context of inflammation. In particular, SARS-CoV-2 entry, replication and disease pathogenesis are discussed. Models to study autophagy and to formulate novel treatment approaches and pharmacological modulation to fight COVID-19 are debated. The SARS-CoV-2—autophagy interplay is presented, revealing the complex dynamics and the molecular machinery of autophagy. The new molecular targets and strategies to treat COVID-19 effectively are envisaged. In conclusion, our finding underline the importance of development new treatment strategies and pharmacological modulation of autophagy to fight COVID-19.

## 1. Introduction

Autophagy, as a process that maintains intracellular homeostasis and acts as a part of the cell’s defense system, is closely related to lysosome function.

The start of autophagy research, crowned by the Nobel Prize in Physiology and Medicine in 1974, was awarded to Christian de Duve for the discovery of lysosomes through cell fractionation in 1955. His finding was based on the identification of the biochemical content of the new organelle [1]. Further on, electron microscopic studies discovered the lysosome and it was described as a morphological entity [2]. The process of the degradation of intracellular components was then detected, showing the presence of irregular shaped vacuoles containing amorphous materials in addition to lysosomes [3]. Thereafter, the application of different autophagy inhibitors revealed a double membrane-bound structure containing a portion of cytoplasm and organelles lacking hydrolytic enzymes, which was termed as the autophagosome. It was consequently noticed as a single membrane structure, coined as the autophagolysosome, presenting different stages of organelle degradation by lysosomal enzymes [4].

Based on these studies, in 1963, Christian de Duve termed the delivery of cytoplasmic materials to lysosomes for degradation as “autophagy” (from the Greek word for self-eating) [5]. Since then, a multitude of morphological and biochemical experimental approaches were developed to example autophagy and its quantitative estimation, which is still difficult to be precisely assess [6].

Later on, another Nobel Prize in Physiology and Medicine was given to Yoshinori Ohsumi in 2016 for his groundbreaking experiments leading to a new paradigm in the understanding of how the cell recycles its content. His work on yeast opened the path of comprehension of the fundamental importance of autophagy in many physiological processes, such as aging, adaptation to starvation, or response to infection. It was proven that mutations in autophagy genes can cause disease and that the autophagic process is involved in several conditions, including cancer and neurological disorders [7,8]. Yeast models helped the discovery of autophagy-related genes and the introduction of the unified system of gene nomenclature, where *ATG* genes were used to name autophagy genes [9,10]. At present, the number of *ATG* genes is around 40, consisting of genes responsible for the core machinery of the autophagosome as well as genes related to selective modes of autophagy [6]. Nowadays, almost all counterparts of yeast Atg proteins are detected in mammals. This evolutionary preservation suggests that the fundamental mechanisms of autophagy were developed and conserved at a very initial step of eukaryotic evolution. 

Accumulating research in the field acknowledges the role of autophagy in immune response by the direct impacts on the activation, proliferation, and differentiation of immune cells [11]. It affects immune signaling by bidirectional regulation of pattern recognition receptors, which bind to molecules derived by microorganisms [12]. It also assists in antigenic processing and presentation by MHC class II molecules [13].

There is growing evidence supporting the involvement of autophagy in various infectious diseases, including coronavirus (CoV) infections. These viruses affect multiple steps of autophagy and autophagy itself also interferes with the viral cycle [14,15].

Similarly to other positive-strand RNA viruses, CoV captures the intracellular membranes of host cells to form double-membrane vesicles, which promote viral RNA synthesis and protect dsRNA from degradation [16,17]. However, the origin of the bilayer membrane, as well as how autophagy is implicated in the inversion of host membranes into double-membrane vesicles, is still debatable [18].

Although autophagy is now an enormously appealing field in biology, its implications in infectious diseases and particularly in SARS-CoV-2 demands interdisciplinary research to elucidate the complex interactions between the virus and the autophagy machinery.

The aim of the present review is to outline the recent knowledge on the interplay between SARS-CoV-2 entry, replication, disease pathogenesis, and autophagy by presenting the molecular mechanisms of lung inflammation and immunity. Finally, the ultimate goal of all basic and clinical research in infectious diseases is to create reliable models for autophagy studies and to formulate novel and reliable treatment strategies and pharmacological modulation to fight COVID-19.

## 2. Autophagy as a Process—Biological Importance and Types of Autophagy

Autophagy is a key, intracellular, evolutionary, conservative, catabolic process in eukaryotes, through which pathological, redundant, or damaged cell components, as proteins and organelles, alongside other macromolecules, are degraded and eliminated. Lysosomes, acidic structures containing hydrolases, and digestive enzymes [19] play a major role in this process. Various noteworthy environmental conditions, such as starvation, growth factor depletion, hormonal and cytokine levels, and infectious agents can induce autophagy in order to maintain cellular homeostasis. Such a mechanism provides energy and nutrients by recycling unessential cytoplasmic components or through exerting a protective role by eliminating pathogenic elements. The biological importance of autophagy in normal development, metabolism, neurodegeneration, and aging and diseases is also well studied [20,21]. Based on the nature of the cargoes and the mode of delivery to lysosomes, different forms of selective or non-selective autophagy are distinguished. In contrast with the non-selective pathway where bulk cytosolic compounds are degraded, in the selective autophagy cargos, mitochondria, ribosomes, peroxisomes, endoplasmic reticulum (ER), lipids, glycogen, and intracellular pathogens (bacteria, viruses) are specifically recognized and tagged for subsequent digestion. Autophagic selectivity is mostly dependent on distinct receptors, most of which have a ubiquitin-binding domain and an LC3-interacting region (LIR).

Currently, there are three major types of autophagy: microautophagy, chaperone-mediated autophagy, and macroautophagy. **Chaperone-mediated autophagy** (CMA) occurs directly on the lysosome and involves the chaperone Hsc70, which recognizes and binds proteins with a KFERQ amino-acid motif. Upon recognition, substrates are directly translocated via a complex including LAMP2A monomers (Lysosomal-associated membrane protein 2 A variant) onto the lysosomal membrane for subsequent degradation. **Microautophagy** also occurs directly on the lysosome, where, unlike CMA, microautophagy non-selectively uptakes and degrades cytosolic material by invagination of the lysosomal membrane. **Macroautophagy** is the only autophagy pathway that, besides the lysosome, involves an additional organelle, the autophagosome. The process is initiated by mTORC1/ lysosome dissociation, triggering phagophore maturation, so that molecules are selectively or unselectively wrapped into a double-membrane autophagosomal structure. Finally, upon fusion, an autolysosome is formed. Among the key macroautophagy protein components are Beclin-1 (BECN1; involved in the promotion of autophagosome formation), the microtubule-associated protein 1 light chain 3B (LC3B; an essential constituent of the autophagosome membrane), and the lysosomal-associated membrane glycoprotein 1 (LAMP1; a primary constituent of the lysosomal membrane) [21]. The great relevance of the selective macroautophagy is the elimination of unwanted, surplus, or damaged organelles, including mitochondria, peroxisomes, lipid droplets, and endoplasmic reticulum, either through mitophagy, pexophagy, lipophagy, or ERphagy, respectively. Xenophagy is a scavenging pathway where foreign organisms, including viruses, that exert a harmful effect on infected cells are instead selectively eliminated by autophagy [22,23].

The complex molecular networks that underlie these distinct autophagic pathways, in the context of viral infections, and in particular, the interplay between autophagy and SARS-CoV-2 that have been the subject of extensive investigation during the COVID-19 pandemic, will be mentioned in the present review.

## 3. Autophagy in Infectious Diseases

The mechanisms underlying autophagy modulation and its pro-viral and antiviral roles have been stated with the COVID pandemic. However, viruses in turn (HSV, EBV, CAV, DENV, HCV, MHV, PV, VZV, and many others) have evolved diverse adaptive strategies to cope with autophagy-driven host defenses, either by blocking or hijacking the autophagy machinery for their own benefit.

### 3.1. Antiviral Functions of Autophagy

It is now well-established that autophagy is not only involved in cellular homeostasis through digestion and recycling of damaged or surplus cytosolic components, but it is also a key element of both the innate and adaptive immune responses to bacteria, viruses, and protozoa. On the one hand, the direct engulfment and degradation of pathogens already contained within phagolysosomes provides the innate branch of immunity with yet another virus clearance mechanism. On the other hand, MHC class II presentation of cytosolic antigens may also be aided by autophagy. That is how this versatile process promotes the adaptive immune system and exerts an antiviral role on several levels [24,25,26].

### 3.2. Blocking of Autophagy by Viruses

Importantly, as one may expect from the years of coevolution between mammalian cells and viruses, the latter have evolved escape mechanisms and are capable of blocking autophagy in various ways. Interestingly, CoVs (including SARS) were described nearly 20 years ago to prevent the fusion of the autophagosome with the lysosome, which, besides survival, provides them with another membrane to replicate on (discussed below) [25,27].

### 3.3. Pro-Viral Role of Autophagy

Autophagy may not only simply be blocked by viruses, but it can play a pro-viral role as well. One of the most well studied examples is when viruses utilize autophagosomal membranes (double-membrane vesicles, DMVs) as scaffolds to replicate on [24]. Critical steps of virion formation, such as the acquisition of the envelope from the cell membrane and viral assembly, may be strongly supported by hijacking autophagy [28]. Concordant with such a scenario, in lipophagy, lipid droplets (LDs), besides serving as assembly points for virion production, may also fuel viral replication [29,30].

In summary, there are multiple aspects of autophagy that can be deregulated by viruses in order to support viral replication, assembly, exocytosis, and immune escape [31].

## 4. Autophagy and COVID-19

### 4.1. Early Autophagy Reprogramming

SARS-CoV-2, the CoV of the severe acute respiratory syndrome, belongs to the genera of *Betacoronaviride.* The SARS-CoV-2 viral genome consists of an almost 30 kb genome and four structural proteins: spike (S), envelope (E), membrane (M), and nucleocapsid (N). In addition to structural proteins, the CoV genomic RNA also encodes six accessory proteins, known as ORFs (Open Reading Frames), and two polyproteins, PP1a and PP1ab, which are further cleaved into 15–16 non-structural proteins (NSPs) [31].

SARS-CoV-2, but not SARS-CoV, is able to induce autophagy. Various of its viral proteins stimulate early autophagy or inhibit late autophagy and autophagic flux, resulting in the accumulation of autophagosomes. These autophagosomes have crucial functions in both viral replication and virion release [32]. The evolution of SARS-CoV-2 made some unique changes, so it is able to manipulate the autophagy pathway and endocytic pathway in such a way to protect viral replication and dampen host innate immunity response promoting its survival. SARS-CoV-2 blocks phagophore formation and the ER-membrane-specific autophagy to preserve viral replication and latter hijacks the autophagy lysosomal pathway to promote a viral exit. Several viral ORF (ORF3, ORF8, ORF10) proteins have an orchestrated influence on ER- and mitotic-specific autophagy manipulation (ORF3, ORF10), while silencing innate immune signaling (ORF8, ORF10), although some of them also activate the pro-inflammatory and death inflammasome pathways as well (ORF3). The SARS-CoV-2 live cycle and internalization pathways are described in great detail in Mironov et al. [33], showing the complexity and the many unresolved issues present when considering the viral entry and the formation of the viral replication complex and double membrane vesicles (DMVs) necessary for its replication. It was suggested that DMVs could fuse with late endosomes and lysosomes, possibly containing members of the SNARE protein family, such as STX17 and SNAP29, which we discuss later.

The host factors involved in the internalization of endocytic cargo and endosomal trafficking/recycling are essential for the entry of SARS-CoV-2 [34,35]. Late-endosomal/lysosomal GTPase Rab7a is involved in the trafficking and degradation of cell membrane receptors through the endo-lysosomal pathway [36]. Through its association with VPS35, Rab7a recruits the retromer complex to late endosomes, where it helps with endosome maturation and cargo export [37]. Rab7a likely promotes the release of the ACE2 receptor from endosomes by interacting with the SAR-CoV-2 NSP7 protein [34,38].

Infection by RNA viruses stimulates the formation of DMVs where the viral RNA replication complex accumulates and commences replication of the viral genome. Multiple studies have demonstrated that autophagy-related Atg family proteins, such as LC3, Atg5, and Atg12, colocalize with these vesicles and viral replication complexes. Furthermore, viral growth has been shown to diminish in autophagy-deficient cells, indicating that autophagosome-like DMV vesicles may facilitate RNA virus replication as DMVs are important for virion maturation and the protection of dsRNA from cytosolic RNases [39]. Ricciardi et al. recently showed that DMVs are tethered to the endoplasmic reticulum (ER) by thin membrane connectors, resembling the viral replication organelle, where NSP3 and NSP4 have generated DMVs, while NSP6, through oligomerization and an amphipathic helix, have zippered the ER membranes to establish the connectors [40]. Recent NSP6 (ΔSGF) mutagenesis in several SARS-CoV-2 VOC were found to have a *gain-of-function,* such as mutant activity, demonstrating a higher ER-zippering activity. The study found NSP6 to play three key roles in SARS-CoV-2 replication, acting as a filter for communication between the replication organelle and the ER, organizing DMV clusters, and mediating contact with lipid droplets for selective refurbishing of DMVs with LD-derived lipids.

Other two very recent studies have shown that SARS-CoV-2 is able to prevent the formation of the early precursors of autophagosomes, referred to as prophgagophores or the hybrid preautophagosomal structure (HyPAS). These structures are hybrids that are actually formed by fusion of RB1CC1/FIP200-containing vesicles (FAK family kinase-interacting protein of 200 kDa) derived from the cis-Golgi, with endosomally-derived ATG16L1 membranes. They are initially LC3 negative and only later become LC3-positive. SARS-CoV-2 NSP6 expression alone or in active viral infection prevented HyPAS from formation, as well as SIGMAR1 antagonist chloroquine [41,42], further suggesting NSP6’s orchestration role in DMV formation and their autophagy interaction. In light of these new findings, it is easier to understand why so many pathogens address the autophagosomal membrane formation as emerging via convergence of secretory and endosomal pathways.

Most likely, in order to hijack the ER membranes to participate in the formation of DMVs, Tan et al. showed that SARS-CoV-2 prevents endoplasmic reticulum autophagy, termed *ER-phagy*, by binding of the ORF8 viral protein to the autophagy receptor p62, which is required to drive particular structures to the autophagy pathway for degradation [43]. This interaction resulted in ORF8/p62 accumulation in the lipid droplets sequestering ER-phagy-important ORF8 interactors, FAM134B and ATL3. This ER-phagy inhibition resulted in ER stress and has also been found to facilitate DMVs’ formation. Furthermore, two proteins, transmembrane protein 41B (TMEM41B) and TMEM106B, are required for successful SARS-CoV-2 infection and were found to be localized in the ER, interacting with the autophagosome-forming “vacuolar membrane protein 1” (VMP1). The lysosomal cell protein recycling enzyme Cathepsin L was also associated with viral infectivity [44].

The early autophagy protein ATG16L, participating in the second phagophore membrane formation contributor, was found to promote a higher susceptibility to SARS-CoV-2 infection, as carriers of the ATG16L1 T300 had an impaired autophagy and an increased ACE2 expression related to that [45].

It has been previously shown for other CoVs, such as mouse hepatitis virus (MHV), that autophagy is required for DMV formation and viral replication, with the crucial involvement of autophagy-related ATG family members, such as LC3, Atg5, and Atg12 [39].

The initiation stage of autophagy is canonically regulated by the ULK1/ATG1 complex (ULK1, ATG13, ATG101, and FIP200 (also known as RB1CC1), downstream of mTORC1, while the nucleation/expansion stage is governed by the ATG14-Beclin-1-hVPS34/PI3K complex and two ubiquitin-like conjugation systems (ATG5-ATG12 and LC3/ATG8). Through a ubiquitin-like conjugating system that employs ATG7 as an E1-like activating enzyme and ATG10 as an E2-like conjugating enzyme, ATG5 and ATG12 undergo conjugation and associate with ATG16L1. The resulting ATG12-ATG5 complex functions as an E3-like enzyme and is vital for the lipidation of ATG8 family proteins, facilitating their attachment to vesicle membranes. Ultimately, the autophagosome fuses with the lysosome to form an autolysosome, which enables the degradation of engulfed contents [46,47].

Recently, it was discovered that the expression of papain-like protease PL(pro), a viral protease of SARS-CoV-2, is sufficient to alter starvation-induced autophagy, possibly by lowering the levels of the ULK1 protein and interfering with the formation of the *autophagy initiation complex* [48]. This action is different from the papain-like protease PLP2 encoded by other CoV, where PLP2 interacts directly with LC3 and Beclin 1 (BCN1), promoting early autophagy [49] and reflecting evolutionary differences in CoV strategies to exploit the autophagy-lysosomal pathway.

Turco et al. [50] showed that ULK1 member RB1CC1/FIP200 orchestrates the cargo receptor SQSTM1/p62 autophagophore engulfment, recruiting the ATG12-ATG5-ATG16L1 complex, where ATG8-mediated RB1CC1 displacement captures non-engulfed cargo. FIP200, previously described as part of the HyPAS, was shown by Wang et al. to restrict RNA virus infection by facilitating Retinoic acid-inducible gene I (RIG-I) receptor activation [51]. RIG-I senses viral RNA and instigates an innate immune signaling cascade to induce type I interferon expression (IFN I). We will later show that this pathway is targeted in multiple ways by SARS-CoV-2, and here, we will consider its relation to the autophagy pathway and its perturbation. Since FIP200 facilitates RIG-I activation, its silencing impairs RIG-I signaling and increases host susceptibility to RNA virus infection.

Ohnstad et al. [52] showed another major role of FIP200 in ATG7-independent, NBR1 cargo-receptor-mediated clustering, where FIP200 bypasses the role of LC3 lipidation in autophagy. In the absence of LC3, TAX1BP1 clusters around the NBR1 receptor and TBK1 are required for autophagosome formation. The absence of autophagy, despite the presence of lipidation machinery, highlights the dual role of mammalian autophagy receptors in not only tethering cargo to autophagic membranes via LC3, but also independently recruiting upstream autophagy factors to initiate local autophagosome formation [52]. This phenomenon is further exploited by RNA viruses, such as SARS-CoV-2, as TBK1 is targeted by ORF10 for both autophagy and IFN I suppression, as we will describe in detail below. Intriguingly, ORF10 interacts with both TBK1- and LC3-mediated protein–protein interactions, while TBK1 participates in the LC3-lacking sequestration pathway. *This suggests that SARS-CoV-2 is indeed unique in its approach to autophagy exploitation reprogramming, as it has adapted to addresses both LC3-dependent and LC3-independent autophagophore formation.*

The entry of SARS-CoV-2 involves cellular membrane structures and activates lipid biosynthetic pathways, specifically class III phosphoinositide 3-kinase (PI3K), also known as VPS34. The latter produces PI3P (phosphatidylinositol 3-phosphate), which is used in many cellular processes, including the autophagy nucleation/expansion stage. A number of studies were conducted to determine whether autophagy is the link between VSP34, SARS-CoV-2 replication, and DMV fragmentation. However, only the VSP34-specific inhibitor VPS34-IN1 was able to inhibit the replication of SARS-CoV-2, while many autophagy-specific inhibitors failed, implying that SARS-CoV-2 exploits VSP34 endocyte trafficking control functions rather than autophagy alone [53,54].

In SARS-CoV-1 infection in vitro studies, ATG5 was found to be important for viral replication [39]. Interestingly, the SARS-CoV-2 virus–autophagy interaction was not shown to implicate activation players of the canonical autophagy pathways BECN1, ATG5, and ATG7 [55]. Beta-coronaviruses were found to replicate via ATG5-dependent but also independent pathways in DVMs that are very close to autophagosome double membrane structures [56]. This observation may be associated with ATG5’s autophagy-independent functions in host responses to viral and bacterial infections. The Atg5-Atg12 conjugate, an essential regulator of autophagy, has been demonstrated to play a significant role in modulating innate, antiviral, immune responses. It has been shown that the Atg5-Atg12 conjugate negatively regulates type I interferon (IFN) production by directly interacting with retinoic acid-inducible gene I (RIG-I) and interferon-beta promoter stimulator 1 (IPS-1) via their caspase recruitment domains (CARDs), consequently promoting RNA virus replication within host cells [57].

In addition to its role in the formation of an interferon gamma-inducing conjugate with ATG12 and/or ATG16L [57,58] in several antiviral responses not related to autophagy machinery engagement (ATG5-ATG12/ATG16L), ATG5 has been found to independently participate in other responses against infection [59]. Calpains were found to mediate some non-autophagy ATG5 activities and calpain-2 was found to facilitate the SARS-CoV-2 spike protein-mediated cell attachment by positively regulating the cell surface levels of ACE2 [60]. A widely observed cellular phenomenon involves calpain-mediated ATG5 cleavage, which is associated with the induction of pro-apoptotic events. Upon cleavage, Atg5 translocates to the mitochondria and interacts with the pro-apoptotic protein Bcl-xL, leading to the release of cytochrome c and the activation of apoptotic caspases [61]. In addition, multiple studies have implicated ATG5 and Beclin-1, along with other autophagy-related genes, in the interplay between autophagy and apoptosis, which was already stated as “autophagic cell death” [62,63]. This form of PCD plays a role in various physiological and pathological contexts, such as development, cancer [61], and neurodegenerative diseases [62].

Wang et al. [64] showed very recently that the loss of Atg5 promotes lysosomal exocytosis and secretion of extracellular vesicles and neutrophils’ excessive degranulation, due to sequestration by an alternative conjugation complex, ATG12-ATG3, of ESCRT protein ALIX, which acts in membrane repair and exosome secretion. All these findings illuminate the potential evolutionary shift towards ATG5-independent SARS-CoV-2 viral replication. Calpains are required for facilitating viral entry, however, they may also promote ATG5-induced apoptosis, which could decrease the likelihood of successful viral replication. Additionally, evading ATG5 dependent autophagy could potentiate viral exit from the cell.

### 4.2. Late-Stage Incomplete Autophagy

SARS-CoV-2 infection alone results in a perturbation of the autophagy-lysosome pathway, manifested by suppression of its specific members. Similarly, major pathways, such as hypoxia-induced HIF-1 signaling and innate antiviral RIG-1 signaling, were also affected [65], intertwined with mitophagy modulation, as we will discuss later. Evidence suggests that CoVs, including SARS-CoV-2, utilize the late endosome/lysosome exocytic pathway for release. The presence of SARS-CoV-2 virions is more pronounced in endosomes that are identified by the late-endosomal/lysosomal marker LAMP1. Additionally, SARS-CoV-2 infection leads to a significant increase in LAMP1 levels [66]. The late-endosomal/lysosomal GTPase Rab7a is involved in endolysosomal maturation and appears to be crucial for viral egress [66].

The late phase of autophagy is marked by the merger of the autophagosome with the lysosome, a process regulated by multiple protein complexes, such as SNARE (STX17-SNAP29-VAMP8), HOPS (homotypic fusion and protein sorting) (VPS39, VPS11), and ATG (ATG14) [67]. The accessory viral proteins are ORF3a [68,69] and ORF7a [70]. They have been shown to prevent the fusion between autophagosomes and lysosomes during late autophagy stages. The HOPS component VPS39 directly interacts with and is sequestered by the ORF3a localized to the late endosome, preventing the HOPS complex from engaging with the STX17 autophagosomal SNARE protein STX17 as well as RAB7. This prevents the STX17-SNAP29-VAMP8 SNARE complex from coming together, which is necessary for fusion with lysosomes. ORF3a promotes the recruitment of the BORC-ARL8b complex and exocytosis-related SNARE proteins to facilitate the retrograde transport of lysosomes and subsequent fusion with the plasma membrane [71]. Lysosomes are similarly affected and rendered less functional by ORF3a expression, becoming deacidified. Lysosomal neutralization promotes their exocytosis.

Infection with SARS-CoV-2 inhibits autophagy, leading to the accumulation of autophagosomes and amphisomes, as well as the late-endosomal sequestration of VPS39 [68,69]. Another SARS-CoV-2 protein, NSP6, affects autophagy flux at the late stage by targeting ATP6AP1 and preventing acidification of the lysosome by blocking pro-cathepsin D cleavage, but not autophagosome-lysosome fusion [72].

**Figure 1:**
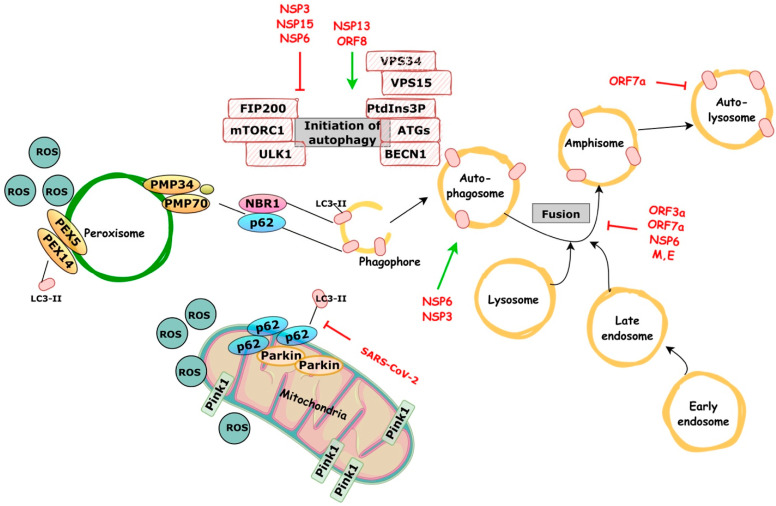
Schematic representation showing SARS-CoV-2 interference in the macroautophagic and microautophagic (mitophagy and pexophagy) pathways.

*Incomplete autophagy* is a dysfunctional self-eating process of intracellular constituents in which accumulating autophagosomes do not fuse with lysosomes for destruction, causing the blockage of autophagic flux. In a normal condition, general autophagy can promote both cell survival and death, but incomplete autophagy is critical to breaking cellular homeostasis and solely promotes cell death [73]. Infection with SARS-CoV-2 results in an incomplete autophagy response, increased autophagosome formation, and deficient maturation. Significantly reduced autophagy caused by genetic knockout of essential autophagic genes limits replication efficiency. ORF3a alone is sufficient to induce complete autophagy. SARS-CoV-2 ORF3a interacts with the UVRAG autophagy regulator to promote the Beclin-1-Vps34-Atg14 complex, but it inhibits the Beclin-1-Vps34-UVRAG complex (promotes phagophore formation but inhibits phagophore maturation). In particular, despite the fact that SARS-CoV ORF3a and SARS-CoV-2 ORF3a share 72.7% sequence homology, the first has no influence on cellular autophagy responses. Thus, these findings provide mechanistic evidence that ORF3a takes over host autophagy machinery to enhance the replication of SARS-CoV-2 and highlights the idea of targeting the autophagic pathway for COVID-19 treatment [74]. SARS-CoV-2 interference in the macroautophagic and microautophagic (mitophagy and pexophagy) pathways is illustrated on Figure 1.

The autophagy-lysosomal pathway is important in the process of SARS-CoV-2 infection; viral proteins interact with it at multiple points. SARS-CoV-2 ORF8 and NSP13 promote autophagic degradation, whereas NSP6, NSP15, and NSP3 inhibit autophagy by impairing autophagosome formation and preventing lysosome acidification. NSP6 can also induce LC3-II-containing vesicles and activate autophagosome formation; however, the size of autophagosomes formed by NSP6 is smaller than that induced by starvation. The ORF3a, ORF7a, M, and E proteins block the fusion of autophagosomes and amphisomes with lysosomes by interacting with the HOPS complex component VPS39. ORF7a decreases lysosome activity and thus inhibits autolysosomal degradation. Mitochondria also play an important role in SARS-CoV-2 clearance; however, SARS-CoV-2 hinders mitophagy by interfering with the binding of p62 to the LC3 protein.

Functionally, the enrichment of the virus in endocytic organelles results in lysosome deacidification, inactivation of lysosomal digestive enzymes, and alteration of antigen presentation. These modifications are linked to a viral unconventional exit, which is a lysosome-dependent exocytosis process regulated by the small Arf-like GTPases, Arl8b and RAB7 [66].

### 4.3. Mitophagy and Innate Immune Responses Reprograming

Mitophagy, as addressed above, is a kind of selective microautophagy that is used to degrade damaged mitochondria. It employs the same basic autophagy machinery (primarily encoded by ATG genes) as other types of selective autophagy, whether induced by different intrinsic signals (e.g., genetically programmed versus cellular metabolism) or extrinsic factors (e.g., environmental stimuli) [75,76].

Mitochondria are intracellular energy factories that produce ATP and participate in cellular processes, including ROS generation, autophagy, and apoptosis. According to emerging data, they play a crucial role in COVID-19 by modulating innate and adaptive immunity as well as viral replication [77,78]. SARS-CoV-2, for example, binds to the translocase of the outer mitochondrial membrane 70 and disrupts the host cell type I IFN response, allowing for viral multiplication [79]. Furthermore, SARS-CoV-2 hijacks host mitochondria to decrease immunity by controlling mitochondrial dynamics, function, respiration, and mitochondrial DNA release, allowing it to avoid host innate immunity. During SARS-CoV-2 infection, mitophagy is initiated by the viral gene products, ORF10 and M, which localize to the mitochondria. This process induces the degradation of MAVS [80] and suppresses antiviral immune responses [81]. Many viruses interact with mitochondrial membranes and components to promote ROS generation, modulating host pathways and viral chemical modifications. Viruses therefore manage the oxidative condition of the host cell to drive viral replication by gradually raising mitochondrial ROS levels. However, during acute viral infections, mitochondrial activity is disrupted and excessive ROS are generated, resulting in host cell harm or death [82].

To maintain mitochondrial homeostasis and to destroy viral RNA, host cells initiate mitophagy via the PINK1-PRKN pathway in response to virus-induced mitochondrial damage. SARS-CoV-2, on the other hand, prevents mitophagy by blocking the binding of p62 to the LC3 protein [83].

Galectin-8, a cytosolic lectin, functions as a pattern and/or danger recognition receptor for intracellular pathogens and mediates selective autophagy (such as mitophagy and xenophagy) in response to viral replication [84,85]. It detects highly glycosylated viral proteins, such as the SARS-CoV-2 spike protein, and initiates antiviral xenophagy or virophagy. Nonetheless, the SARS-CoV-2-encoded 3CLpro protease targets and cleaves galectin-8 and the adaptor FYCO1, which in turn, impedes recruitment of the autophagy adaptor NDP52 to compromised endosomes. This disruption of xenophagy enables the virus to circumvent antiviral autophagy [86]. Recent findings have demonstrated that various variants of concern possess mutations in multiple regions of the M-protein. These mutations do not impact the protein’s primary protease activities related to viral replication; however, they exhibit significant variability in their secondary activity to cleave galectin-8. Since galectin-8 is also involved in cytokine and chemokine secretion and potentially contributes to the development of cytokine storms [87], these variants could promote varying levels of TNF-α/IL-6 expression in both PBMC culture and CD14+ monocytes and B cells, thereby compromising the host’s immune response [88].

Li et al. [81] have found that ORF10 marks mitochondrial antiviral signaling proteins (MAVS) for degradation using mitophagy modulation: ORF10 binds mitophagy receptor Nip3-like protein X (NIX) so it can translocate to the mitochondria, where it facilitates the interaction of NIX with LC3B. Mitophagy activation drives MAVS degradation, resulting in type I interferon (IFN-I) genes’ suppression. Silencing NIX is capable of abrogating all three: mitophagy activation and IFN-I via MAVS suppression.

SARS-CoV-2 infection induces mitochondrial dysfunction, leading to the release of mitochondrial DNA (mtDNA), and promotes the formation of syncytia, which results in the transport of chromatin and micronuclei from the nucleus to the cytoplasm; this anomalous cytoplasmic DNA triggers an immune response by activating DNA pattern recognition receptor cyclic GMP-AMP synthase (cGAS), subsequently leading to cGAS-STING-mediated IRF3-type I interferon (IFN) and autophagy-mediated antiviral responses [89,90]. In their study, Su et al. [91] discovered a novel function of ORF3a in SARS-CoV-2, which is not shared by SARS-CoV. Specifically, ORF3a was found to act as a selective disruptor of the STING-LC3 complex, thereby inhibiting cGAS-STING-induced autophagy and facilitating viral replication, while leaving the induction of IRF3-type I IFN unaffected. Replication controlling SARS-CoV-2 3-chymotrypsin-like cysteine protease (3CLpro) was also shown to disrupt the assembly of the STING functional complex by inhibiting its K63-ubiquitin modification [92].

Han et al. [93] have shown that ORF10 acts in concert with ORF3a in regard to cGAS-STING, as overexpression of ORF10 inhibits cGAS–STING-induced interferon regulatory factor 3 phosphorylation, translocation, and subsequent IFN induction. Mechanistically, ORF10 interacts with STING, attenuates the STING–TBK1 association, and impairs STING oligomerization and aggregation and STING-mediated autophagy; ORF10 also prevents the endoplasmic reticulum (ER)-to-Golgi trafficking of STING by anchoring STING in the ER. As we have previously seen, Ohnstad et al. [52] have demonstrated that this would impair LC3-independent autophagophore formation. ORF3a was found to lack RIG-I rec eptor (RLR) inhibitory functions, but it was able to block the nuclear accumulation of p65 and inhibit nuclear factor-κB signaling. SARS-CoV-2 structural protein N plays the role of a unique RLR inhibitor, thus working in concert with ORF3a [92].

SARS-CoV-2 viral proteins, ORF6 and NSP13, induce degradation of the DNA damage response kinase CHK1 via the proteasome and autophagy pathways, respectively. The loss of CHK1 results in a dNTP deficiency, impairing S-phase progression, inducing DNA damage, activating pro-inflammatory pathways, and promoting cellular senescence. SARS-CoV-2 N-protein impairs 53BP1 focal recruitment by interfering with damage-induced, long, non-coding RNAs, thereby reducing DNA repair. SARS-CoV-2 promotes its replication at the expense of dNTPs by increasing ribonucleoside triphosphate levels and hijacks damage-induced, long, non-coding RNA biology, posing a threat to genome integrity, triggering an altered DNA damage response activation, inflammation, and cellular senescence [94]. This process is supported by reprogrammed early autophagy and incomplete late autophagy, which impairs damaged organelle clearance control in cells.

SARS-CoV-2 infection has been shown to disrupt antigen presentation, leading to decreased protective immunity and increased inflammatory responses in infected cells. The role of autophagy in SARS-CoV-2 infection has been described as “double-edged” due to its dualistic effects [95]. Some aspects of autophagy flux are enhanced by SARS-CoV-2 infection, such as ORF3a promoting lysosomal function and exocytosis for viral release or ORF8 directly interacting with MHC class I proteins to suppress antigen presentation via the autophagy-lysosome pathway [71,96]. However, the early autophagy process is hijacked to promote viral replication, while many autophagy receptor pathways and innate immune signaling methods are downmodulated. *We suggest that the effect of SARS-CoV-2 on autophagy can be better explained as a bifurcative disruption rather than as a dualistic role.*

### 4.4. Putative Pexophagy Involvement

**Pexophagy** (specific microautopagy) selectively targets excess or incompetent single membrane peroxisome organelles for degradation [19] through the ubiquitin-mediated p62/NBR1 receptor system [97,98]. Other mechanisms for peroxisome degradation exist but are less prominent. Peroxisomes play critical roles in cellular metabolism, redox homeostasis, and immune signaling, and their abundance can adjust to meet changing metabolic needs [99]. Viral infections, such as by enveloped viruses, can induce peroxisome expansion to support viral replication [100]. Cellular cargo might be transported for autophagy, independently of ubiquitin status, through protein–protein interaction motifs, ubiquitin-like modifiers, and sugar or lipid-based signaling. FAM134 proteins directly interact with LC3/GABARAP proteins to deliver fragmented ER structures to autophagosomes [19,101]. LC3-II competes with PEX5 for binding to PEX14, which could be a quality control mechanism to regulate peroxisome abundance [102,103]. SARS-CoV-2 may interfere with pexophagy by inhibiting the binding of p62 to LC3, or NBR1 on peroxisomes, thus increasing ROS and oxidized metabolites and triggering inflammasome activation, cytokine production, and cell death. The exact mechanisms by which SARS-CoV-2 interferes with pexophagy and its implications are still being studied.

### 4.5. Treatment and Pharmacological Modulation

COVID-19 disease complications lead to the development of acute respiratory distress syndrome, followed by lung failure and eventually, death [104,105]. Unfortunately, there are no approved COVID-19-specific drugs for prevention or treatment. According to the WHO, around 58% of the drugs pending for clinical trials affect autophagy [106]. Although these drugs differ in their mechanism of action, the accumulation of autophagosomes after treatment switches to apoptosis in virally infected cells [107,108]. Among the first anti-SARS-CoV-2 drugs applied in clinical practice is **chloroquine.** It is primarily used for treatment of malarial and autoimmune diseases, but it also exerts antiviral properties [109,110]. Chloroquine decreases the terminal glycosylation of ACE2, which acts as a cellular receptor of SARS-CoV, thus inhibiting the entry of the virus [111]. At the same time, the levels of ACE2 on the host cell surface remain unchanged. Additionally, chloroquine at a concentration of 6.9 μM abolishes SARS-CoV infection in vitro in Vero E6 cells (EC_90_) [112] and decreases the number of SARS-CoV antigen-bearing cells [111,113,114]. Mauthe et al. have reported that chloroquine inhibits autophagy by interfering with autophagosome fusion with lysosomes rather than dealing with the degradative activity of this organelle [115].

**Ruxolitinib** represents another anti-SARS-CoV-2 drug that selectively acts as a JAK1/2 inhibitor. It is used predominantly in multiple myeloma and in other hematological malignancies, such as myelofibrosis [116,117]. The application of ruxolitinib induces autophagy in acute myeloid leukaemia cells through the downregulation of c-Myc, MCL-1, and BCL-xL, followed by the inhibition of the entire mTORC1/S6K/4EBP1 pathway [118]. Upon treatment, ruxolitinib leads to autophagosome formation in ARH-77 and NCI-BL 2171 cells compared with non-treated cells [116].

Two other antiviral drugs, **lopinavir and ritonavir**, block HIV aspartate protease, which is an important enzyme involved in intracellular HIV assembly [119]. As a result, immature virions are formed that are unable to infect new cells [120]. Lopinavir/ritonavir are also able to inhibit SARS-CoV 3CL^pro^ in vitro. However, the required dose for achievement of an in vivo effect in humans is weakly tolerable [121]. Even though, clinical trials with lopinavir/ritonavir have been initiated soon after the outbreak of the COVID-19 pandemic. It has been found that the efficiency of lopinavir/ritonavir treatment is increased when combined early (five days after the onset of COVID-19 symptoms) with IFN beta-1b and ribavirin. As a result, the recovery time is reduced from twelve to seven days [122]. The application of lopinavir alone or in combination with ritonavir on both primary mouse and human adipocytes resulted in activation of an endoplasmic reticulum stress response. This is followed by inhibition of autophagy activity, cell differentiation, and induced cell apoptosis [123].

Being a part of the innate immune response, **type I** IFN **(IFN-I)** represents an important antiviral factor [124]. The data available in the literature shows that autophagy affects the expression of both IFN-I and IFN-I-receptors and thus regulates IFN-I responses. Subsequently, clinical trials with IFN-α 2a/2b have been set to treat severe forms of COVID-19 [124]. The treatment of HepG2 cells with IFN-α2b for 48h triggers the accumulation of markers for autophagy, such as Beclin1 and LC3-II proteins, as well as single- or double-membrane vacuoles containing intact and degraded cellular organelles [125]. The exposure of microglia on combined antiretroviral therapy that includes **tenofovir disoproxil fumarate**, **emtricitabine, and dolutegravir** resulted in lysosomal membrane permeabilization accompanied by loss of lysosomal function, increased pH, and decreased cathepsin D activity [126]. Moreover, the lysosomes were unable to fuse with newly produced autophagosomes, suggesting dysregulated autophagy and increased neuroinflammation. Interestingly, the simultaneous treatment of aged HIV-positive patients with tenofovir disoproxil fumarate and emtricitabine lowered COVID-19 severity [127].

The use of **corticosteroids** is strongly recommended in the protocols for COVID-19 treatment, especially for severe cases and those with COVID-related acute respiratory distress syndrome [128,129,130]. As a result, a reduction in COVID-19 mortality and in non-COVID-19 acute respiratory distress syndrome patients (2740 patients in 16 trials) has been observed (RR 0.82, 95% CI 0.72–0.95, ARR 8.0%, 95% CI 2.2–12.5%, moderate certainty) [131]. As it was shown in the cases of infection of human monocytes with spores of the airborne fungal pathogen *Aspergillus fumigatus,* corticosteroids lead to selective transfer of the autophagy protein LC3 II in phagosomes [132]. They also block autophagy in vivo and ex vivo by LC3 II recruitment in *Aspergillus fumigatus* phagosomes via targeting syk kinase phosphorylation and further, ROS production [132].

The data generated from transcriptomics and lipidomics studies showed drastic alterations of lipids, such as plasmalogens, in infected Vero E6 cells, correlating with enhanced virus replication. The application of **niclosamide (NIC),** a drug used for COVID-19 treatment with very well known anti-helminthic properties significantly decreases the total lipid profile. More specifically, Garrett et al. [133] demonstrated that NIC treatment significantly reduces the required for-COVID-19-replication plasmalogens, diacylglycerides, and ceramides.

**Figure 2:**
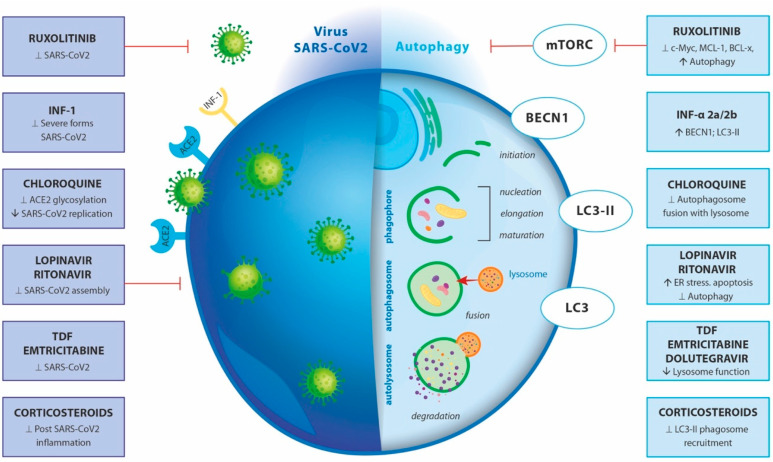
Effect of different drugs on SARS-CoV-2 and autophagy. The left part of the figure presents SARS-CoV-2 entry and replication in an infected cell and the effect of the enlisted drugs; The right part represents the effect of the enlisted drugs on the autophagy machinery in the cell. ↑—indicates activation/enhancement; ⊥—indicates repression/inhibition, ↓—indicates deactivation/degeneration.

The effects of different drugs on SARS-CoV-2 and autophagy are demonstrated in Figure 2.

### 4.6. Adverse Effects of Autophagy-Targeting Drugs

The lack of specific modulation on COVID-19 of autophagy-related drugs is the reason for the development of severe side effects upon systemic application that are mostly due to off-target activity. For example, the accumulation of damaged mitochondria due to impaired mitophagy after chloroquine treatment results in renal tubular dysfunction [129]. Chloroquine-induced nephrotoxicity in distal tubular cells has been caused through autophagy-dependent and autophagy-independent mechanisms, including interplay with the production and signaling of cyclic adenosine monophosphate [130]. Other observed side effects include retinopathy, gastrointestinal effects, cardiomyopathy, and myopathy [131,132].

**Emtricitabine and enofovir** are drugs that cause complications in the kidneys of transplant orthotopic liver patients, leading to reversible acute renal failure and acute tubular necrosis [133]. The use of JAK-inhibitor agents, such as ruxolitinib in patients with myelofibrosis, results in adverse effects, such as anaemia, cytopenias, weight gain, and an increased risk of opportunistic infections [134]. After comparison of the side effects of different antiretroviral regimens among 137 patients, Kim et al. found that 16 out of 35 (45.7%) developed skin rash, 7 out of 35 (20%) had gastrointestinal discomfort or pain, 7 out of 35 (20%) had diarrhea, 6 out of 35 (17.1%) had hyperbilirubinemia, and 3 out of 35 (8.5%) had headache or dizziness [135]. Retinopathy is a frequently developed complication in patients receiving IFN alfa-2b therapy [136]. In addition to that, flu-like symptoms, nausea, anorexia, depression, confusion, myalgia, fatigue, joint pain, and neuropsychopathy can be observed [137]. The prolonged intake of oral corticosteroids leads to increased bone resorption and decreased bone formation in a dose-dependent manner. The use of inhaled corticosteroids is also linked to higher chances for fracture risk [138]. From these findings, it can be concluded that there is an urgent necessity to develop drugs that specifically target SARS-CoV-2 and the autophagy pathway.

## 5. Models to Study Autophagy in Infections

The autophagic process is quite complicated and involves numerous actors. The studies of autophagy rely on static and dynamic methodologies targeted at tracking the primary components of autophagy, such as autophagosomes, lysosomes, and autophagolysosome morphology. They are also focused on formation dynamics, autophagy flux, and other autophagy-mediated outcomes. The second alternative perspective on autophagy is to investigate these processes through various model systems, both in vitro and in vivo. The paper “*Guidelines for the use and interpretation of assays for monitoring autophagy*”, produced every three years, is the most comprehensive resource on techniques and models to research autophagy in health and disease, acting as a compendium covering all methods and models reported thus far [134]. As for models to study COVID-19, there are some very detailed reviews on the subject, such as the ones of Rosa et al. [135], on both in vitro based systems as well as on animal models. The ones by Sun et al. and Kang et al. on lung and brain microphysiological systems are accordingly dedicated to the study of COVID-19 [136,137]. There are several models that are commonly used to examine the modulation of autophagy due to a COVID-19 infection. Some of these include:

**Cell culture models**: autophagy modulation in response to SARS-CoV-2 infection can be investigated in vitro using human lung cells, such as A549 or Calu-3 cells. **A549 cells** are human lung epithelial cells used in several studies as a model of lung injury during COVID-19 and autophagy modulation. SARS-CoV-2 9b induces an increased LC3-II/LC3-I ratio in A549 cells, which is ATG5-dependent and correlates with an increased number of LC3-positive autophagosomes [138]. SARS CoV ORF9b interacts with the mitophagy regulator, TOMM70, to cause mitochondrial dysfunction in A549 cells, potentially affecting non-selective autophagy [139]. **Calu-3 cells**: Calu-3 cells are human bronchial epithelial cells used to investigate autophagy modulation in response to SARS-CoV-2 infection. For example, SARS-CoV-2 was found to replicate only in Vero, Vero E6, and Calu-3 cells, and was confirmed with real-time RT-PCR. Images from TEM revealed multiplication of the virus in various vesicles of these cells, accompanied by apoptosis of the host cells. In cell culture studies, human embryonic kidney cells (**HEK 293T**) are extensively employed. Thus, in a recent study to elucidate autophagy interactors encoded by the SARS-CoV-2 genome, all cell lines described thus far (Calu-3, HEK293T, and Vero E6) were used and 28 viral proteins were screened. SARS-CoV-2 caused an incomplete autophagy response, impaired maturation, and declined autophagy by ORF3a alone [140]. In the previous investigation, HEK 293T cells were transfected with ORF3a to follow the SARS-CoV-2 ORF3a- autophagy pathway interactions. Additionally, human-induced pluripotent stem cells (**iPSCs**) are successfully used as a model when primary cells or explants are not readily available. Thus, human iPSC-CMs (cardiomyocytes) have also been adopted to test the efficacy of the autophagy inhibitor bafilomycin, which significantly reduced the number of infected cells [141].

**Animal models**: In vivo animal models, such as mice or rats, can be used to study the effect of SARS-CoV-2 infection on autophagy. These models can also be utilized to examine the function of autophagy in the pathophysiology of COVID-19 and to evaluate possible treatment agents. **Mice**: In the case of SARS-CoV-2, as the virus does not directly infect mice, transgenic mouse models are used. Transgenic mice are widely used to study the modulation of autophagy in response to SARS-CoV-2 infection. Female hACE2 transgenic mice (C57BL/B6 background) were infected with a viable SARS-CoV-2 virus, and the effects on autophagy were observed and measured. The transgenic mice express hACE2, which was driven by the mouse ACE2 promoter, as described previously [142]. Autophagy inhibition suppressed SARS-CoV-2 replication and ameliorated viral-induced pneumonia in this hACE2 transgenic mouse. Similarly, in a xenografted fetal human lung male mice model (NOD/ShiLtJGpt-Prkdcem26Cd52Il2rgem26Cd22), bearing a fragment of human fetal lung in the dorsal subcutaneous space [143], autophagy inhibition also suppressed SARS-CoV-2 replication and induced viral pneumonia in xenografted human lung tissues [144]. The human xenograft turned out to be a very capable lung model for such studies. SARS-CoV-2 affects cellular metabolism and autophagy, restricting its spread. Infected cells accumulate critical metabolites, activate autophagy inhibitors, and reduce proteins involved in autophagy induction, membrane nucleation, and phagophore assembly. The autophagy markers LC3B-II and P62, which are integrated into phagophores, accumulate in a **Syrian hamster model [46]**.

Miniature 3-dimensional structures called 3D organoids, derived from stem cells, can mimic the structure and function of different tissues, including the lung. Human normal colon organoids derived from benign portions of colorectal cancer resection tissue were used to demonstrate that polyamine supplementation and autophagy activation could effectively regulate cellular metabolism and inhibit SARS-CoV-2 proliferation [145].

**Microphysiological models,** also known as **“organ-on-a-chip” models**, are small devices that contain microfluidic channels and tissues or cells that mimic the structure and function of different organs, including the lung. These models allow for the controlled delivery of the virus and the monitoring of its effects on cellular processes. These models offer several advantages over traditional cell culture and animal models. They provide a more controlled and reproducible environment for studying the effects of the virus on autophagy and other cellular processes. They better mimic the microenvironment and cellular interactions that occur in vivo. Human organoids and organ-on-chip models have been employed for COVID-19 research due to the species-specificity of infections, notably in the brain, where no animal model properly recreated nervous system damage caused by SARS-CoV-2. Except in transgenic ACE2 mouse models, where animals perished from brain injury, which is not related to genuine human illness, monkey and mouse models exhibited no alterations [136].

It is important to note that each of these models has its own advantages and limitations and that a combination of models is often used to gain a more comprehensive understanding of the effects of SARS-CoV-2 infection on autophagy. 

## 6. Conclusions

At present, it is well defined that autophagy is a fundamental process, just as basic to cells’ life as synthesis is. Further research will elucidate the significance of autophagy to numerous physiological and pathological conditions. There are loads of questions that demand answers, especially concerning process dynamics and the development of novel robust systems and new devices allowing the envisagement of the molecular machinery of autophagy. There are still plenty of mysteries associated with the complex SARS CoV-2–autophagy interactions to be uncovered. Only then, we could justly understand the molecular targets, which we can aim to, in our efforts, effectively prevent and treat COVID-19.

## Abbreviation

***a***	
A549	human lung cells
ACE2	angiotensin-converting enzyme 2
AKT-mTOR signaling	protein kinase B-mammalian target of rapamycin complex
APCs	antigen presenting cells
Arl8b	Arf-like GTPases
ARP2/3	actin-related protein 2/3 complex
ARH-77	B lymphoblast cell line
ASC	adaptor apoptosis-associated speck-like protein with a caspase-1 recruitment domain
*ATG*	autophagy-related genes
ATP6AP1	S1 subunit of the enzyme V-type proton ATPase.
***b***	
BCL-xL	B-cell lymphoma-extra large
BECN1(Beclin-1)	mammalian ortholog of the yeast autophagy-related gene 6
BORC-ARL8b	complex multisubunit complex- regulates lysosome positioning
***c***	
Calu-3	human bronchial epithelial cells
CARD	caspase recruitment domain
CatB/L	cathepsin B and L
CAV	Coxsackie viruses
c-Myc	proto-oncogene
CNS	central nervous system
CXCR-4	C-X-C chemokine receptor type 4
***d***	
3D organoids	miniature 3-dimensional structures that can mimic the structure and function of different tissues
DAMPs	damage-associated molecular patterns
DENV	Dengue virus
DMV	double-membrane vesicles
Dolutegravir	antiretroviral medication
***e***	
EBV	Epstein Bar virus
eIF2a	translation elongation factor
Emtricitabine	nucleoside reverse-transcriptase inhibitor
ER	endoplasmic reticulum
***f***	
FAM134	reticulon protein- ER-phagy receptor
***g***	
GABARAP	GABA type receptor-associated protein
GSDMD	gasdermin D
GTPase	hydrolase enzyme binding nucleotide guanosine triphosphate
***h***	
HCV	hepatitis C virus
HEK 293T	human embryonic kidney cells
HIF-1	hypoxia-induced transcription factor-1
Hsc70	heat shock cognate 71 kDa protein
HSV	herpes simplex virus type I
HOPS	homotypic fusion and protein sorting
***i***	
ICP34.5	infected cell protein 34.5
IFN	interferon
iPSCs	human induced pluripotent stem cells
***j***	
JAK1/2	Janus kinase- (tyrosine kinases)
***k***	
KSHV	Kaposi’s sarcoma-associated herpes virus
***l***	
LAMP1	lysosomal associated membrane glycoprotein 1
LAMP2A	lysosomal associated membrane protein 2
LC3	microtubule associated protein 1 light chain 3B
LIR	LC3-interacting region
Lopinavir	antiretroviral protease inhibitor
***m***	
MAVS	mitochondrial antiviral-signaling protein
MCL-1	induced myeloid leukemia cell differentiation protein
MHC	major histocompatibility complex
MHV	murine hepatitis virus
mTORC1/S6K/4EBP1	mammalian target of rapamycin complex 1/p70 ribosomal S6 kinases/4E-binding protein 1
***n***	
NBR1	neighbor of BRCA1 gene
NCI-BL 2171	small cell lung carcinoma
NDP52	calcium binding and coiled-coil domain 2
NF-kB	nuclear factor-κB (transcription factor)
NIC	niclosamide
NK	natural killer cells
NLRP3	NLR family pyrin domain containing 3 inflammasome
NS4B	membrane-associated protein involved in viral replication or assembly
NSPs	non-structural proteins
***o***	
OPTN	Organ Procurement and Transplantation Network
ORFs	Open Reading Frames
ORF3a	encodes a viral accessory protein
ORF8	encodes viral accessory protein, Betacoronavirus NS8 protein
***p***	
p62	SQSTM1(sequestosome 1)- ubiquitin-binding scaffold protein
PAMPs	pathogen-associated molecular patterns
PCD	programmed cell death
PEX3	peroxisomal Biogenesis Factor
PI3K	phosphoinositide 3-kinase, also known as VPS34.
PI3P	phosphatidylinositol 3-phosphate
PINK1	PTEN induced kinase 1
PKR	protein kinase R
PMP	peroxisome membrane protein
PP1a	protein phosphatase 1
PRKN	Parkin RBR E3 ubiquitin protein ligase
PTEN	fosfatidilinositol-3,4,5-trisfosfato 3-fosfatasa
PV	Poliovirus
PYCARD	apoptosis-associated speck-like protein containing a CARD (or ASC)
PYD	pyrin domain
***r***	
Rab-7a	Ras-related protein late-endosomal / lysosomal GTPase
RCD	regulated cell death
RHIM	homotypic interaction motif
Ribavirin	antiretroviral medication inhibits- RNA/DNA viruses
RIP	proteins containing a receptor-interacting protein
Ritonavir	antiretroviral medication, protease inhibitors
RNP	ribonucleoprotein complexes
ROS	reactive oxygen species
RTC	replication and transcription complex
Ruxolitinib	Janus kinase inhibitor
***s***	
SMVs	single-membrane vesicles
SNAP	soluble *N*-ethylmaleimidesSensitive factor attachment proteins
SNAP29	synaptosome associated protein 29, SNARE protein, in autophagy
SNARE	SNAP receptor
SNX27	sorting nexin family member 27
STX17	Syntaxin 17, autophagosomal SNARE protein
***t***	
TAK1	transforming growth factor beta-activated kinase 1
Tax1BP1	Tax1-binding protein 1
TGN	trans-Golgi network
TNFR1	tumor necrosis factor receptor 1
TMEM41B	transmembrane protein 41B
TMPRSS2	transmembrane serine 2 protease
Tenofovir DF	tenofovir disoproxil fumarate, HIV medication
***v***	
VAMP8	vesicle-associated membrane protein 8
Vero E6	epithelial cell from African green monkey
Viroporin	small hydrophobic multifunctional viral proteins
VMP1	vacuolar membrane protein 1
VPS	vacuolar protein sorting proteins (as VPS26, VPS29 and VPS35)
Vps34	class III phosphoinositide 3-kinase (PI3K)
VZV	Varicella zoster virus
***w***	
WHO	World Health Organization
***z***	
ZBP1	Z-DNA binding protein
